# Postoperative *Nocardia* Endophthalmitis and the Challenge of Managing with Intravitreal Amikacin

**DOI:** 10.1155/2016/2365945

**Published:** 2016-03-13

**Authors:** Sikander A. K. Lodhi, G. Ashok Kumar Reddy, C. Aruna Sunder

**Affiliations:** ^1^Sarojini Devi Eye Hospital, Hyderabad 500028, India; ^2^GHR Micro Diagnostics, Dwarakapuri Colony, Hyderabad 500082, India

## Abstract

*Nocardia* is a rare cause of delayed onset postoperative endophthalmitis after cataract surgery and it usually carries a guarded visual prognosis.* Purpose*. To highlight the clinical presentation, microbiological profile, and treatment outcome in a case of nocardial endophthalmitis after manual small incision cataract surgery.* Methods*. This case report highlights the typical features of* Nocardia* endophthalmitis, which presented six weeks after undergoing small incision cataract surgery. The case was managed by pars plana vitrectomy with intravitreal antibiotics. Intravitreal amikacin was used based on microbiologic work-up.* Results*. The endophthalmitis part was controlled but the case developed amikacin induced macular infarction which jeopardized a good visual outcome.* Conclusion*.* Nocardia* endophthalmitis manifests late after cataract surgery in an aggressive manner and carries a poor visual prognosis. An early diagnosis and the use of correct antibiotic regimen may salvage the vision. But the present case shows that one should always be wary of potential retinal toxicity with intravitreal amikacin.

## 1. Introduction

*Nocardia *are aerobic, Gram-positive, weakly acid fast filamentous bacteria and are a rare cause of endophthalmitis. Postoperative nocardial endophthalmitis is a delayed onset infection after cataract surgery and it usually carries a guarded visual prognosis. Nocardial endophthalmitis can occur in both immunocompromised and immunocompetent hosts. Most of these cases are reported from south India [1–5].

We report a case of postoperative* Nocardia* endophthalmitis in an immunocompetent individual. The infection was successfully managed by vitrectomy and intravitreal antibiotics but the case developed retinal toxicity due to intravitreal aminoglycoside antibiotic.

## 2. Case Report

**Figure 1:**
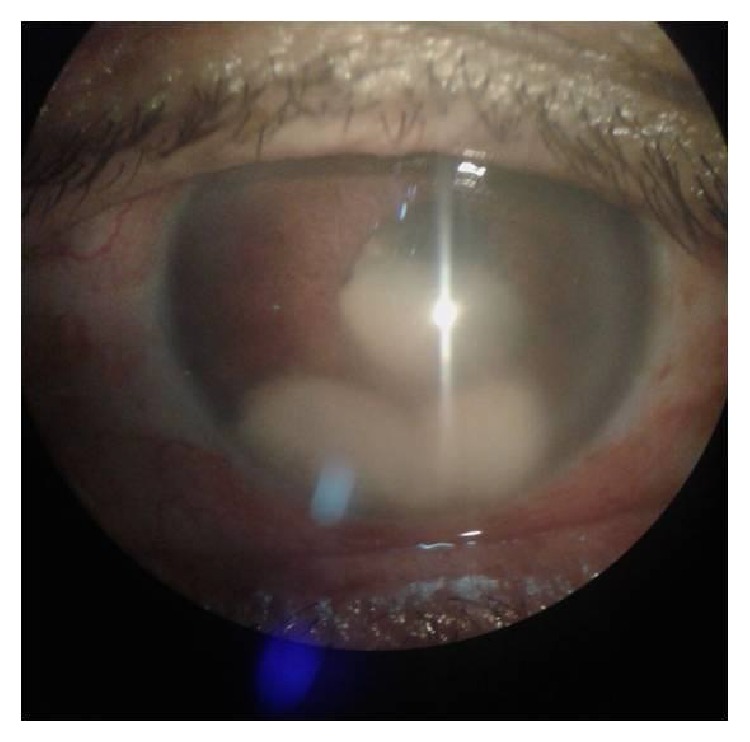
Fluffy white exudates in anterior chamber and surrounding the IOL in capsular bag.

**Figure 2:**
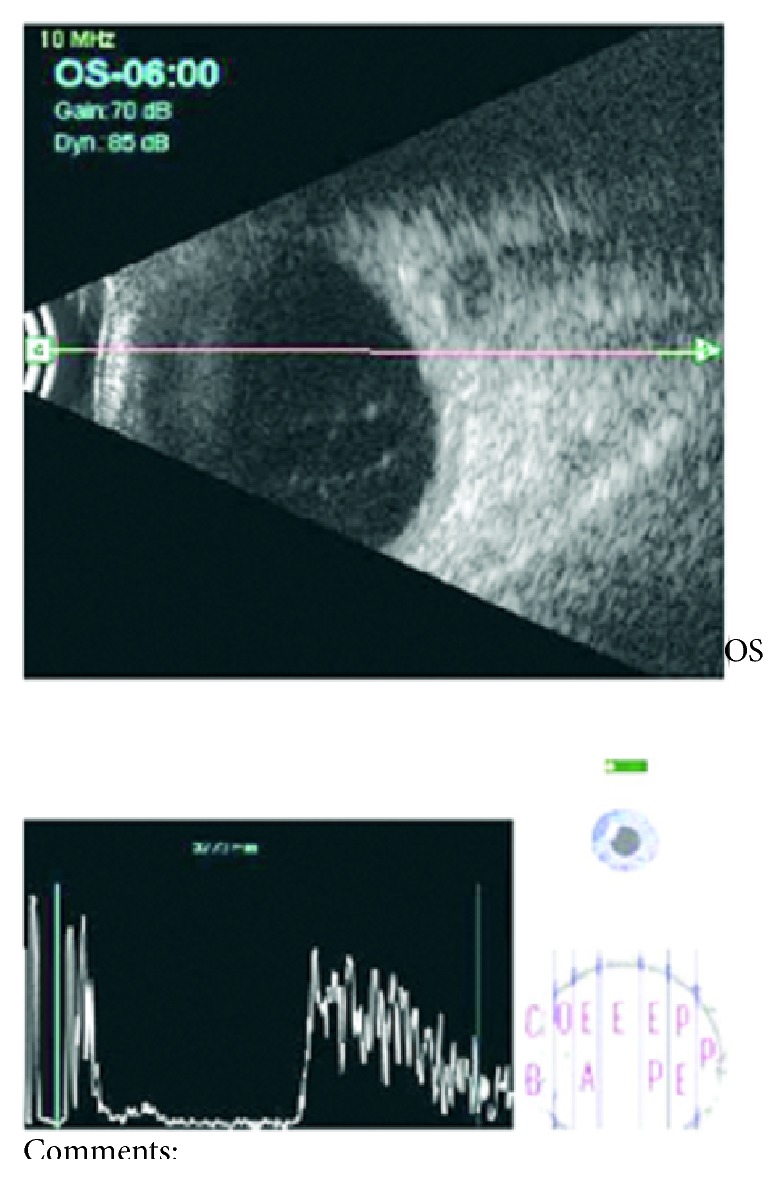
Ultrasound showing fine vitreous opacities with flat retina.

A fifty-year-old female patient presented to us 45 days after an uneventful manual small incision cataract surgery. The patient was from a farming background and otherwise she did not have any health issues and she was not a diabetic. She gave history of mild pain, redness, and blurred vision in the operated eye (left eye) for five days. On examination, visual acuity in the right eye was 6/6 and in the left eye hand movements were perceived close to face. On slit lamp examination, left eye had corneal epithelial edema and fluffy white cotton ball-like exudates in the anterior chamber and the intraocular lens was surrounded by the same exudates in the capsular bag (Figure 1). Intraocular pressure was normal. Ultrasound of the eye showed fine vitreous opacities of low reflectivity with flat retina (Figure 2).

**Figure 3:**
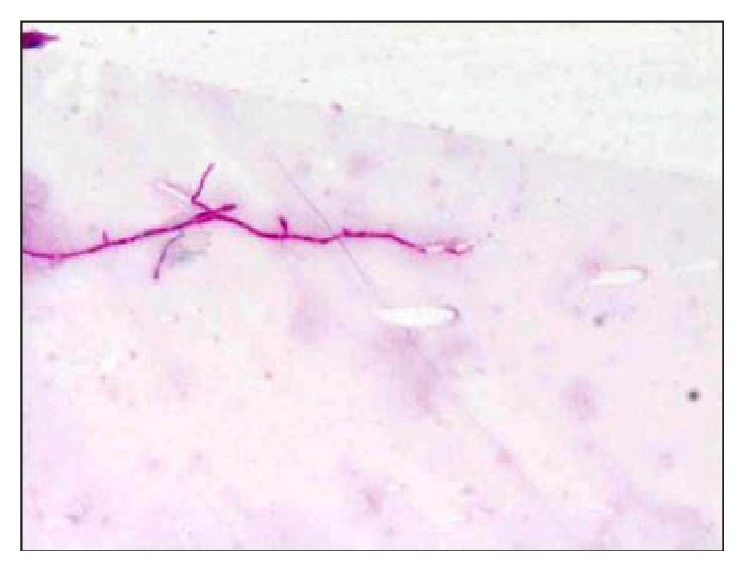
Gram-positive thin long beaded branching filaments of* Nocardia.*

**Figure 4:**
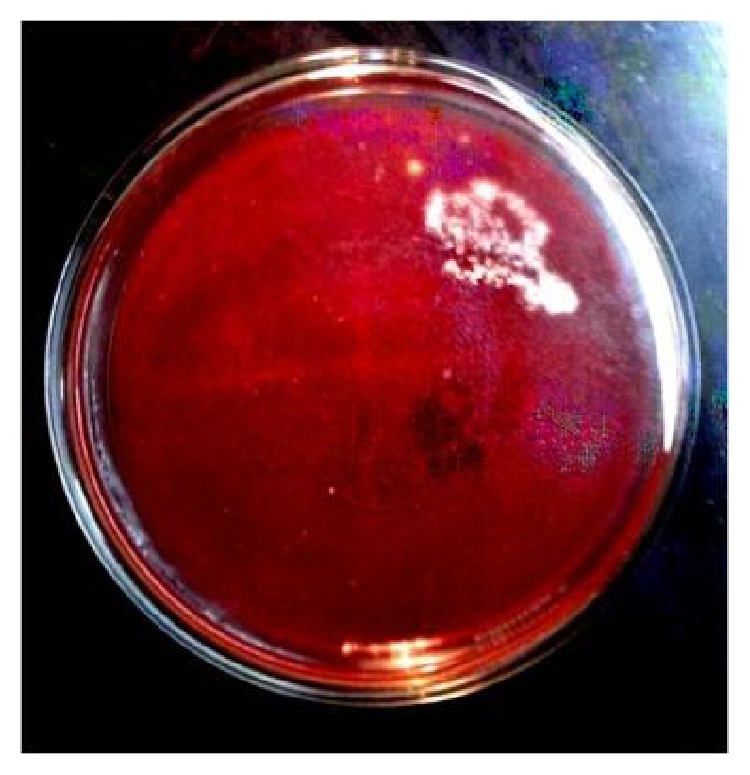
Dry, raised, chalky white colonies of* Nocardia* on blood agar.

Because of delayed onset and the type of exudates in the anterior chamber, fungal endophthalmitis was a strong suspicion. Pars plana vitrectomy with anterior chamber wash was done and intravitreal vancomycin, ceftazidime, and voriconazole were given. Direct microscopic examination of vitreous and anterior chamber fluid showed Gram-positive thin long beaded branching filaments and acid fast thin long filaments in modified acid fast staining (Kinyoun stain) (Figure 3). On blood agar dry, raised, chalky white colonies were seen after 2 days of incubation (Figure 4). The colonies were further identified as* Nocardia asteroids* based on colony morphology and biochemical reactions. The isolate was susceptible to amikacin, gentamicin, ciprofloxacin, ofloxacin, gatifloxacin, moxifloxacin, and co-trimoxazole.

**Figure 5:**
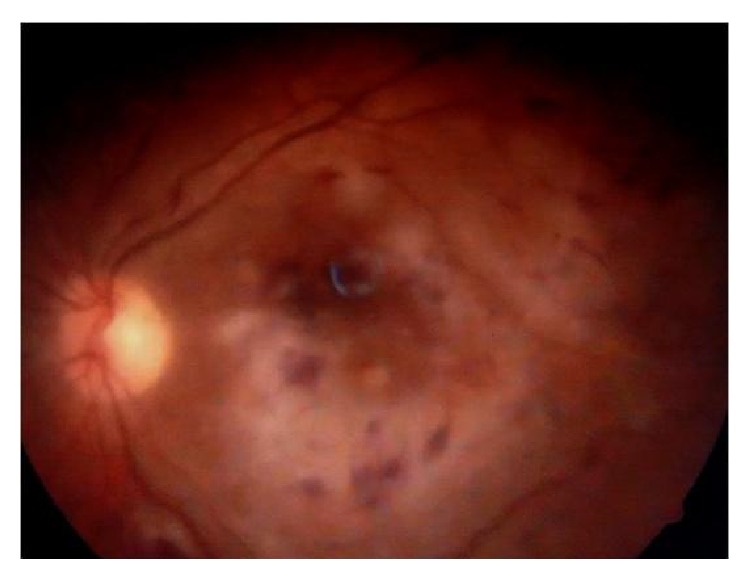
Cloudy swelling, cotton wool spots, and scattered intraretinal haemorrhages.

**Figure 6:**
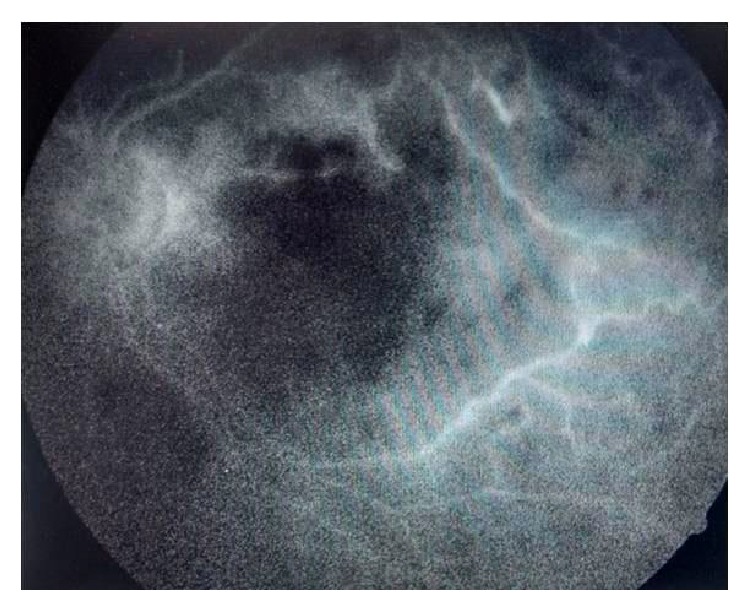
Fluorescein angiogram showing macular arteriolar occlusion with staining of the arteriolar walls along superior and inferior arcades.

**Figure 7:**
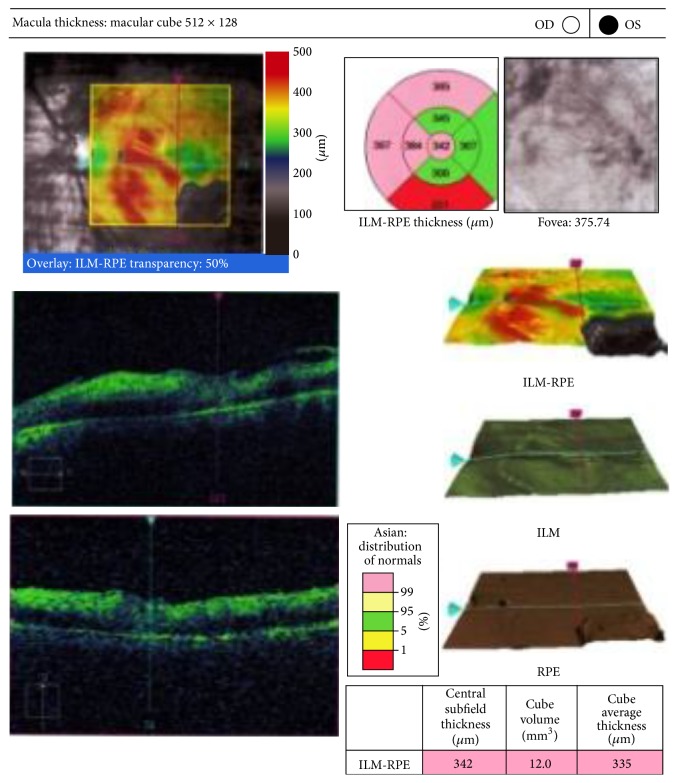
OCT showing increased macular thickness.

Depending on the sensitivity report, intravitreal amikacin (200 *μ*g/0.1 mL) was given. The dose of intravitreal amikacin was halved because it was a vitrectomised eye. Topical gatifloxacin and gentamicin eye drops were continued. Systemically, tablet co-trimoxazole was started and given for 6 weeks. Two weeks later, vision had improved to counting fingers at one meter. Anterior segment was quiet, mildly hazy vitreous and the macula was pale and cloudy looking with multiple intraretinal hemorrhages (Figure 5). Amikacin induced macular infarction was suspected. Fluorescein angiography showed multiple capillary nonperfusion areas (Figure 6). The OCT showed increased macular thickness (central foveal thickness 342 *μ*m), hyperreflectivity of inner retinal layers corresponding to areas of retinal edema (Figure 7).

**Figure 8:**
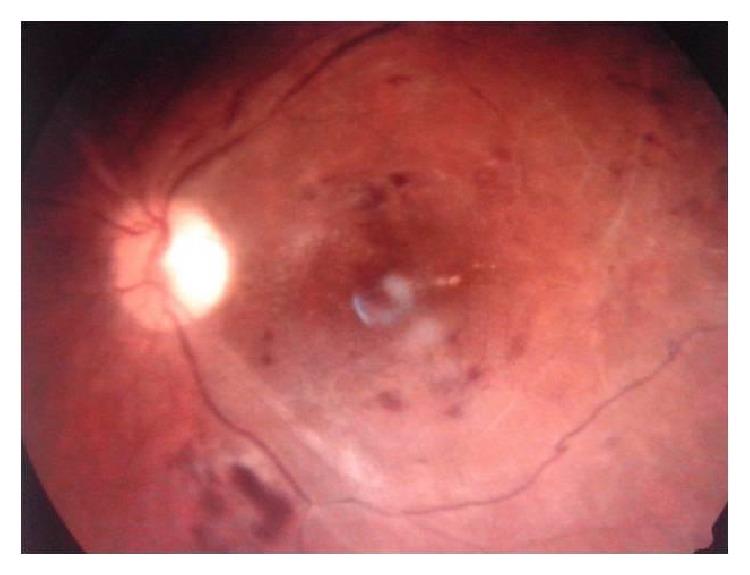
Macular edema subsided.

**Figure 9:**
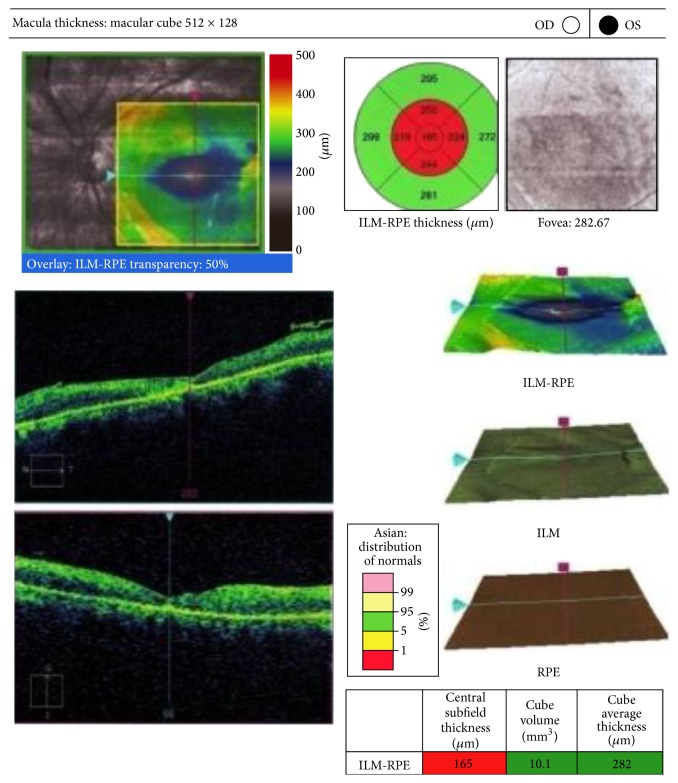
Hyperdense and adherent RPE and retinal layers.

To treat this ischemia and inflammation induced macular edema, intravitreal steroids could not be used in the backdrop of endophthalmitis. Intravitreal Bevacizumab was used for its anti-inflammatory properties. The vision improved to counting fingers at 2 meters and the macular edema subsided (Figure 8). On OCT, the macular thickness had decreased but the differentiation of different retinal layers was not evident and foveal centre showed hyper dense and adherent RPE and retinal layers (Figure 9). After follow-up of more than three months now, the condition of the eye is stable.

## 3. Discussion

There are two large case series of postoperative* Nocardia* endophthalmitis reported from South India [1, 4]. Decroos et al. [1] from Hyderabad, India, presented 16 cases of culture proven postoperative* Nocardia* endophthalmitis. In this series, 75% of patients presented with distinctive anterior chamber findings, such as the exudates on corneal endothelium or nodule-like deposits at the pupillary border. Final visual outcome was poor (final mean BCVA 2.1- ± 0.89 logMAR units.). Haripriya et al. [4], from Madurai, India, reported 24 culture proven cases of exogenous nocardial endophthalmitis following cataract surgery. Cotton ball exudates, fluffy exudates on corneal endothelium, and nodular exudates on iris and hypopyon were the anterior segment findings. Visual outcome was poor in most patients with 79% (19/24) of patients obtaining a final visual acuity of hand motions or worse. In both these studies, the intense anterior chamber involvement was in sharp contrast to minimal vitritis seen. Both these studies reported highest antibiotic sensitivity for amikacin (90% and 87.5%). In our case, the presentation of endophthalmitis was six weeks after cataract surgery. The anterior segment involvement was intense and comparatively vitreous exudates were minimal. Following vitrectomy and intravitreal amikacin (with a reduced dosage of 200 *μ*g/0.1 mL), the infection part was controlled but the visual outcome was not satisfactory due to development of macular infarction. The rationale for amikacin use was based on the sensitivity report and the results of the previous case series [1, 4]. Previous reports have shown that amikacin is a frequently chosen option for* Nocardia* infections [5–7].

Though macular ischemia is a rare complication following intravitreal amikacin injection, it is reported both following vitrectomy with intravitreal injection and following intravitreal injection alone [8–10]. Only one case of macular toxicity, possibly due to amikacin, was reported out of 420 cases in Endophthalmitis Vitrectomy Study [11]. Several explanations have been put forth to explain the amikacin induced retinal toxicity: dilution errors, increased intraocular pressure following intravitreal injection, and variation in the liquefaction status of vitreous humor. The reason for macular predilection could be the contact of the drug with macula in supine position. Individual susceptibility may also be a factor. It is recommended that amikacin be better avoided when other antibiotics are sensitive to the organism. Amikacin may be used with a calculated risk when other antibiotics are resistant.
